# RE: Comment On: Vonoprazan on the Eradication of *Helicobacter pylori* Infection

**DOI:** 10.5152/tjg.2023.231302

**Published:** 2023-04-01

**Authors:** Jiaming Huang, Ye Lin

**Affiliations:** 1 Department of Gastroenterology, Ganzhou People’s Hospital, Jiangxi, China

Dear editor,

I have read the letter^1^ commenting on my article.

There are many antibiotics used for eradiaction of *Helicobacter pylori* (*H. Pylori*), such as metronidazole, levofloxacin, clarithromycin, amoxicilin and furazolidone. Metronidazole, levofloxacin and clarithromycin have high resistance rate in China, amoxicilin and furazolidone are more commonly used for eradiaction of *H. pylori*, although furozalidone is not a commonly used drug in Europe and Turkey in the first line. The other reason that furazolidone was chosen was because of its cheap price.

In the study, the treatments were given as first line.

Esomeprazole was used with a dose of 2 × 20 mg in traditionla quadruple therapy group in the present study, as esomeprazole 2 × 20 mg was recommended by 2022 Chinese National Clinical Practice Guideline on *H. pylori* eradication treatment.^2^

Though it would be better to make a baseline upper gastrointestinal system endoscopy to see if there was peptic ulcer or another complication of *H. pylori*, not all participants would like to accept upper gastrointestinal system endoscopy, not only because its price, but also because it is discomfortable.
